# Cyanobacteria-Based Bio-Oxygen Pump Promoting Hypoxia-Resistant Photodynamic Therapy

**DOI:** 10.3389/fbioe.2020.00237

**Published:** 2020-03-24

**Authors:** Tao Sun, Yingying Zhang, Chaonan Zhang, Hanjie Wang, Huizhuo Pan, Jing Liu, Zhixiang Li, Lei Chen, Jin Chang, Weiwen Zhang

**Affiliations:** ^1^Center for Biosafety Research and Strategy, Tianjin University, Tianjin, China; ^2^Frontier Science Center for Synthetic Biology and Key Laboratory of Systems Bioengineering, Ministry of Education of China, Tianjin, China; ^3^School of Life Sciences, Tianjin University, Tianjin, China; ^4^Laboratory of Synthetic Microbiology, School of Chemical Engineering and Technology, Tianjin University, Tianjin, China; ^5^Collaborative Innovation Center of Chemical Science and Engineering, Tianjin, China

**Keywords:** oxygenic cyanobacteria, hypoxia, photodynamic therapy, indocyanine green, nanoparticles, injectable hydrogels

## Abstract

Hypoxia not only alters tumor microenvironment but leads to the tumor progression and metastasis as well as drug resistance. As a promising strategy, photodynamic therapy (PDT) can inhibit tumor by catalyzing O_2_ to cytotoxic reactive oxygen species. However, its effects were limited by hypoxia and in turn deteriorate hypoxia due to O_2_ consumption. Hereon, aiming to alleviate hypoxia and promote PDT, a bio-oxygen pump was created based on cyanobacteria, which are the only prokaryotic organisms performing oxygenic photosynthesis. Detailly, controlled-release PDT via loading indocyanine green into mesoporous silica nanoparticles was established. Then bio-oxygen pump based on a fast-growing cyanobacterium *Synechococcus elongatus* UTEX 2973 was tested and further packaged together with PDT to create an injectable hydrogel. The packaged hydrogel showed stable oxygen production and synergetic therapy effect especially toward hypoxia 4T1 cells *in vitro*. More importantly, strong *in vivo* therapeutic effect reaching almost 100% inhibition on tumor tissues was realized using PDT equipped with oxygen pump, with only negligible *in vivo* side effect on healthy mice from *S. elongatus* UTEX 2973. The new photo-oxygen-dynamic therapy presented here provided a promising strategy against hypoxia-resistant tumor and may worth further modifications for therapeutic application.

## Introduction

Cancer is a leading cause of death worldwide, accounting for an estimated 9.6 million deaths in 2018 (Bray et al., 2018). For several decades, various strategies targeting oncotherapy have been developed and evaluated (Dolmans et al., 2003; Gorrini et al., 2013; Desterro et al., 2019). Among them, photodynamic therapy (PDT), which contains two individually non-toxic components, i.e., photosensitizer and light illumination with specific wavelength to transfers energy from light to molecular O_2_ to generate cytotoxic reactive oxygen species (ROS), has been used for more than 100 years (Dolmans et al., 2003). Nevertheless, a common feature of most tumors is the low oxygen level called hypoxia, which leads to the progression, metastasis, radiation and drug resistance of cancer cells, as well as the altering tumor microenvironment (Harris, 2002; Muz et al., 2015). Meanwhile, effect of PDT has been found restricted by hypoxia due to the limited oxygen available and its conversion of O_2_ to ROS may in turn deteriorate the hypoxia degree. Hence the improvement of oxygen availability in tumor tissues could not only alter the tumor microenvironment but also enhance the PDT therapeutic effect.

Cyanobacteria are the only prokaryote microorganism capable of oxygenic photosynthesis via respectively taking sunlight and CO_2_ as the sole energy and carbon source (Stanier and Bazine, 1977). Besides their roles as primary producers on earth (Giordano et al., 2005), cyanobacteria have been considered as model organisms for photosynthesis research and even “photosynthetic cell factories” to produce renewable chemicals (Lea-Smith et al., 2015; Gao et al., 2016). More interestingly and excitingly, biomedical applications based on cyanobacteria have been evaluated in recent years (Raja et al., 2016). For example, Cohen et al. (2017) presented a novel system that rescued the myocardium from acute ischemia using photosynthesis through intramyocardial delivery of the model cyanobacterium *Synechococcus elongatus* PCC 7942 (Cohen et al., 2017). In addition, Yin et al. (2019) reported the role of *S. elongatus* PCC 7942 in accelerating cutaneous wound healing by secreting extracellular vesicles to promote angiogenesis (Yin et al., 2019). All these studies demonstrated the promising therapeutic effects of *S. elongatus* PCC 7942. In 2015, *Synechococcus elongatus* UTEX 2973 (hereafter *S.* 2973), whose genome only contained 55 single nucleotide polymorphisms and insertion-deletions compared to that of *S. elongatus* PCC 7942 (Yu et al., 2015), was isolated and demonstrated preferable properties like fast growing and high light tolerance. More importantly, as optimized growing temperature of *S.* 2973 was similar to that of human body, it may represent a more robust and promising chassis for biomedical applications than *S. elongatus* PCC 7942.

**FIGURE 1:**
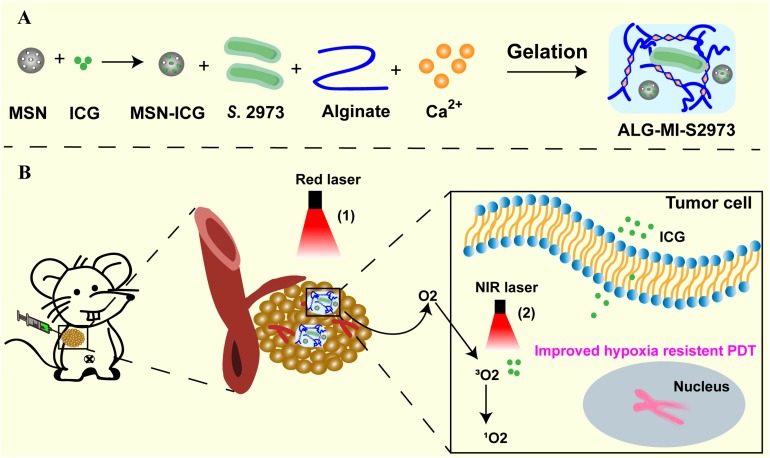
Schematic illustration of the photo-oxygen-dynamic therapy. **(A)** *S.* 2973 and MSN loaded with photosensitizer ICG were mixed with sodium alginate to make injectable hydrogels (ALG-MI-S2973). **(B)** The hydrogels ALG-MI-S2973 was intratumorally injected and then formed gels at the tumor site. The tumor tissue was exposed to 640 nm laser to support survival of *S*. 2973 to generate O_2_, which alleviated the hypoxia microenvironment and further enhanced PDT effects. After releasing, ICG excited by 808 nm laser could exert efficient PDT effects to inhibit tumor growth. MSN, mesoporous silica nanoparticles; ICG, indocyanine green; ALG, hydrogels based on sodium alginate; MI, ICG-loaded MSN; ALG-MI-S2973, hydrogels packaging MSN-ICG and *S*. 2973.

In this study, aiming to simultaneously alleviate the tumor hypoxia and the effect of PDT, a photo-oxygen-dynamic therapy (PODT) strategy combined of a light-driven bio-oxygen pump based on the fast-growing cyanobacterium *S.* 2973 and PDT was developed and investigated (Figure 1). Firstly, the controlled-release PDT via loading indocyanine green (ICG) as photosensitizer into mesoporous silica nanoparticles (MSN) was established and investigated *in vitro*. Then, bio-oxygen pump using *S.* 2973 was developed and further packaged together with PDT to create an injectable hydrogel. The *in vivo* biosafety of *S.* 2973 was further measured and the PODT effect was evaluated using a 4T1 tumor model. Excitingly, the packaged hydrogel showed strong *in vivo* therapeutic effect on tumor tissues. The photo-oxygen-dynamic oncotherapy presented here provided a promising strategy against hypoxia-resistant tumor cells and worth further optimization for therapeutic application as mature genetic tools have been developed for *S.* 2973.

## Materials and Methods

### Materials

Indocyanine green (ICG), cetyltrimethylammonium bromide (CTAB), tetraethylorthosilicate (TEOS), diethanolamine and sodium alginate were purchased from Aladdin Bio-Chem Technology Co., Ltd. (Shanghai, China). Propidium iodide (PI), calcein-AM, Hoechst 33528 were obtained from Sigma-Aldrich (MO, United States). IL-2, IL-6, IFN-β and IFN-γ detection kits were bought from H-Y Biological Co., Ltd. (Wuhan, China). CCK-8 cell proliferation and cytotoxicity assay kit and Annexin V-FITC apoptosis detection kit was both obtained from Solarbio Technology Co., Ltd. (Beijing, China).

Cyanobacterium *S.* 2973 was cultivated with BG11 liquid medium (pH = 7.5) under a light intensity of ∼100 μmol photons m^–2^ s^–1^ in an illuminating shaking incubator (HNYC-202T, Honour, Tianjin, China) at 130 rpm and 37°C (Li et al., 2018). 4T1 cells were cultured in RPMI-1640 medium (Gibco) supplement with 10% fetal bovine serum (FBS) and maintained at 37°C, 5% CO_2_ in a humidified atmosphere. Female BALB/c mice (about 20 g) were bought from SPF Biotechnology Co., Ltd (Beijing, China) and maintained in common environment. All animal experiments were operated following the Guidance Suggestions for the Care and Use of Laboratory Animals and principle of 3Rs. All animals were subjected to an anesthesia experiment with a small animal anesthesia machine (Ruiwode Life Technology Co., Ltd., Shenzhen, China). All animals were sacrificed by spine dislocation, and the bodies were chilled at −20°C and uniformly incinerated by the animal center.

### Cell Density, Absorption Spectrum and Dissolved O_2_ Measurement

Cell density of *S.* 2973 was measured at OD_750 nm_ using a UV-2450 spectrophotometer (Shimadzu, Kyoto, Japan). Besides, the images of *S.* 2973, ALG-S2973 and ALG-MI-S2973 with or without red laser irradiation were took to study the effect of 640 nm red light on the activity of *S*. 2973.

Absorption spectrum of *S.* 2973 was investigated to evaluate the viability of *S.* 2973 in different conditions using the spectrophotometer. To identify the optimal power intensity of 640 nm laser, *S.* 2973 were exposed to the irradiation dose of 0, 0.1, 0.25, and 0.5 W/cm^2^ for 5 min. After that, the irradiation time for 1, 5, and 10 min under the same irradiation dose of 0.25 W/cm^2^ was also evaluated, respectively.

To evaluate the elimination of *S*. 2973 by ICG, strains were incubated with ICG of different concentrations (0, 5, 10, and 20 μg/mL). First of all, *S*. 2973 were irradiated with 0.25 W/cm^2^ of 640 nm laser for 5 min in order to generate oxygen. Then the cells were exposed to 808 nm laser with the power intensity of 2.0 W/cm^2^ for 5 min. At last, the absorptions spectrum and images of *S*. 2973 with different treatments were recorded. Dissolved O_2_ was detected using a Dissolved Oxygen Meter (SMART SENSOR, Guangdong, China).

### AM/PI Staining

AM/PI staining was utilized to evaluate the cytotoxic effect of *S.* 2973, ICG and MI against 4T1 cells. The effect of *S*. 2973 on cellular activity was determined under the irradiation dose of 0.25 W/cm^2^. 4T1 cells were seeded in 48-well plate and cultivated in normoxic or hypoxic condition. Cells were treated with ICG and MI (equivalence to 20 μg/mL of ICG) with or without 808 nm irradiation for 24 h. After removing the medium, 1 μM Calcein-AM and PI were added and then observed by inverted fluorescence microscope. The live/dead staining assay was also conducted for 4T1 cells treated with ALG-MI-S2973 and respectively irradiated with only 640 nm laser for 5 min, only 808 nm laser for 5 min or both of them.

### Preparation and Characterization of MSN and Hydrogels

For preparation of MSN-ICG, MSN were synthesized first according to our previous studies (Zheng et al., 2016). In order to load ICG, 40 mg of MSN were re-dispersed with ddH_2_O and stirred with 5 mg of ICG for 12 h in dark. After the reaction, the product was obtained by centrifugation and washing. For preparation of sodium alginate gel co-loaded with MSN-ICG and cyanobacteria (ALG-MI-S2973), ALG aqueous solution was mixed with Glucono delta-lactone (GDL) using a vortex mixer. And then MSN-ICG and *S.* 2973 were added and stirred to mix evenly. PBS containing 1.8 mM Ca^2+^ was injected to form gel.

For characterization of nanoparticles, transmission electron microscope (TEM) images were acquired by JEOL JEM100CXII at operating voltage of 100 kV. The particle size distribution was measured using dynamic light scattering (DLS) on a Zetasizer Analyzer (Malvern). UV-Vis spectroscopic absorbance was captured on a UV-2450 spectrophotometer.

### The *in vitro* Release of ICG

The dialysis bag diffusion technique was used to measure the *in vitro* release of ICG from MSN-ICG and ALG-MI-S2973. A total of 4 mg of MSN-ICG and ALG-MI-S2973 was dispersed in 1 mL ddH_2_O and then put into a dialysis bag. At certain times, 200 μL of the samples was collected to measure the absorbance at 780 nm. The cumulative release of ICG was calculated according to ICG calibration curve.

### Detection of Reactive Oxygen Species (ROS) and Hypoxia

4T1 cells were grown in normoxic condition and incubated with ICG and MSN-ICG containing 20 μg/mL ICG for 24 h. Then the cells were exposed to 808 nm irradiation for 5 min. Finally, cells were stained with hypoxia/ROS kits and observed using a confocal laser scanning microscope. Similar analysis was also performed for 4T1 cells treated with ALG-MI-S2973 and respectively irradiated with 640 nm laser, 808 nm laser or both of them.

### CCK-8 Proliferation Assay

The biosafety of *S*. 2973 on 4T1 cells were determined by CCK-8 assay. Briefly, 4T1 cells were seeded in 96-well plate and cultured in normoxic condition. Cells were co-cultured with *S*. 2973 and irradiated with 640 nm laser. After that cells were treated with CCK-8 reagent (10 μL/well) at 37°C for 2 h. At last, the absorbance at 450 nm was measured by Thermo Synergy HT Microplate Reader.

### Cell Apoptosis Analysis

For analysis of apoptosis, 4T1 cells were seeded in 6-well plate and cultured in normoxic or hypoxic condition. Cells were treated with ICG and MSN-ICG (equivalence to 20 μg/mL of ICG) with or without 808 nm irradiation for 24 h. Then cells were stained with 5 μL of Annexin V-FITC and PI for 15 min in the dark and then subjected to flow cytometry. Cells treated with ALG-MI-S2973 and respectively irradiated with 640 nm laser, 808 nm laser or both of them were also tested by flow cytometry to discuss the cell apoptosis induced by the PDT effect of ALG-MI-S2973.

### The Biosafety Investigation of *S*. 2973

Female BALB/c mice were randomly divided into four groups and subcutaneously injected with *S*. 2973 and ALG-S2973. After 1, 4-, and 7-days’ injection, blood was collected and analyzed with ELISA kits (H-Y biological) according to the instructions. Besides, blood was collected on the 1st, 7th, and 14th day of treatment to conduct blood routine examination. In the end, mice were sacrificed and main organs (heart, liver, spleen, lung, and kidney) were collected for pathological analysis.

### *In vivo* PDT Effects

Each female BALB/c mouse was subcutaneously injected 2 × 10^7^ 4T1 cells into the axilla. When the tumor volume reached 50–100 mm^3^, mice were randomly divided into four groups. ALG-MI and ALG-MI-S2973 (equivalence to 20 μg/mL ICG) were intratumorally injected at 0 day. Tumors were irradiated with Red laser (0.25 W/cm^2^, 5 min) for 3 days. 808 nm laser was used on the 4th day. The body weight and tumor volume were measured every 3 days. After 21 days’ treatment, mice of all groups were sacrificed and then tumors and main organs were collected to study the pathological morphology. Besides, TUNEL staining and HIF-1α analysis were carried out to assess the apoptosis and hypoxia induced by PDT effect of ALG-MI-S2973.

### Histopathology TUNEL and Immunohistochemical Analysis

Main organs of mice were fixed and embedded in paraffin blocks. The sections were stained with hematoxylin and eosin (H&E) and examined by a microscope. TUNEL analysis was used to evaluate the cell apoptosis in tumor tissues. And then the tumor sections were immune-stained with a rat anti-mouse HIF-1α protein and observed by a microscope.

## Results

### Establishment and Investigation of the Controlled-Release PDT System

**FIGURE 2:**
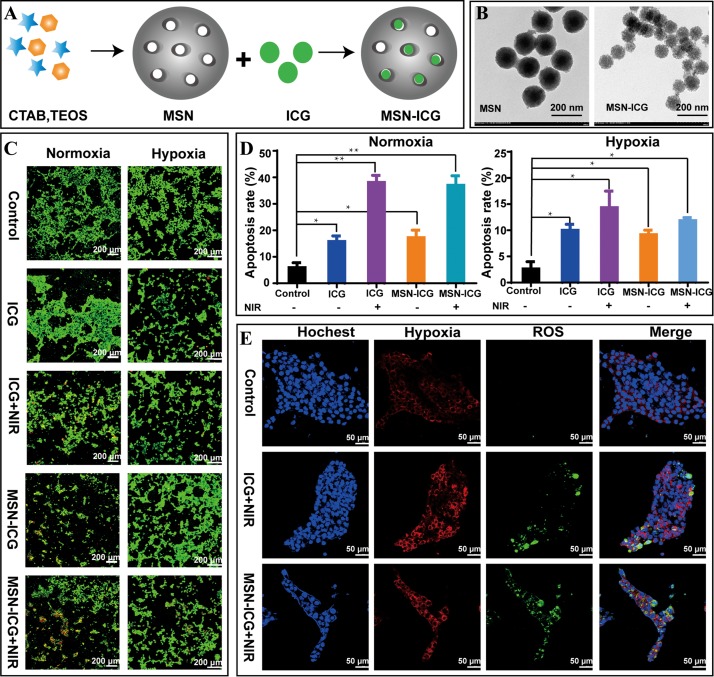
The preparation and characterization of MSN-ICG. **(A)** The preparation process of MSN-ICG. **(B)** TEM images of MSN before and after ICG loading. **(C,D)** Live/dead staining and cell apoptosis analysis of 4T1 cells treated with control, ICG or MSN-ICG with or without 808 nm laser irradiation under normoxic or hypoxic condition. **(E)** Measurements of hypoxia and ROS for 4T1 cells treated with control, ICG or MSN-ICG with 808 nm laser irradiation under normoxia. * *p* < 0.05, ** *p* < 0.01 compared with control group by Student’s *t*-test. MSN-ICG, indocyanine green-loaded mesoporous silica nanoparticles.

To construct the PDT system (Figure 2A), we utilized a near-infrared fluorescent dye ICG that has been approved by the Food and Drug Administration of United States for various clinical applications (Bradley and Barr, 1968; Sauda et al., 1986). Meanwhile, we used near-infrared light to ensure relatively strong penetrability toward tissues and to avoid killing *S.* 2973 when used visible light to support the *in vivo* survival of *S.* 2973. To decrease the injection frequency and prolong the functional time of ICG when used *in vivo*, we established a controlled-release system MSN-ICG by loading ICG into MSN (Table 1). TEM images of MSN before and after ICG loading was shown in Figure 2B and Supplementary Figure S1, suggesting the good uniformity of the nanoparticle. In addition, continuous release of ICG from MSN as long as 24 h could be detected (Supplementary Figure S2). To evaluate the PDT effect, 4T1 cells were treated with control, ICG or MSN-ICG with or without 808 nm laser (NIR) irradiation under both normoxia and hypoxia conditions. As illustrated in Figures 2C,D, the controlled-release MSN-ICG system reached similar PDT effects as ICG alone after irradiation, resulting in ∼37% apoptosis of 4T1 cells under normoxia, which indicated the feasibility of PDT as well as the controlled-release system. In addition, the PDT effect was also demonstrated by the ROS measurement (Figure 2E). On the contrary, the apoptosis rate was dramatically decreased to below 12%∼14% under hypoxia (Figure 2D), suggesting the low oxygen content seriously restricted the PDT. Meanwhile, for 4T1 cells under normoxia, the hypoxia measurement revealed that PDT in turn aggravated hypoxia due to the consumption of O_2_ (Figure 2E).

**TABLE 1:** Detailed characteristics of the biomaterials used in this study.

Name	Description
ICG	Indocyanine green directly for PDT
*S.* 2973	Wild type *S*. 2973 for oxygen generation
MSN-ICG	Nanoparticles via loading indocyanine green into mesoporous silica
ALG-S2973	Hydrogels via packaging *S*. 2973 with sodium alginate
ALG-MI	Hydrogels via packaging MSN-ICG with sodium alginate
ALG-MI-S2973	Hydrogels via packaging MSN-ICG and *S*. 2973 with sodium alginate

### Evaluation of the Bio-Oxygen Pump Based on Cyanobacteria

**FIGURE 3:**
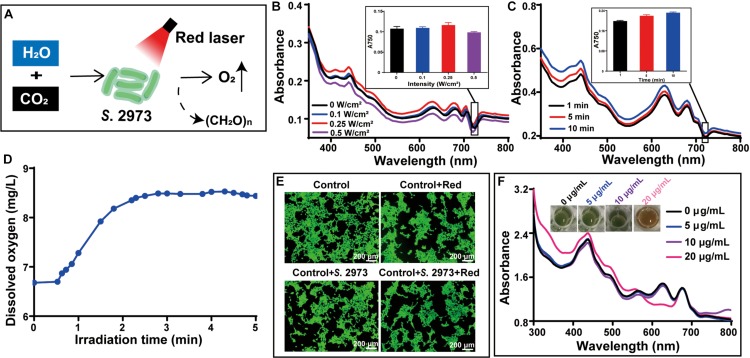
Construction and characterization of the bio-oxygen pump *in vitro*. **(A)** Scheme of oxygen generation system based on *S.* 2973 under 640 nm red laser irradiation. **(B)** Absorption spectrum and OD_750 nm_ of *S.* 2973 irradiated using 640 nm laser with different intensities. **(C)** Absorption spectrum and OD_750 nm_ of *S.* 2973 irradiated using 640 nm laser with 0.25 W/cm^2^ for different times. **(D)** Oxygen evolution of *S.* 2973 under 640 nm red laser irradiation. **(E)** Cell viability and AM/PI staining of 4T1 cells with or without treatments (i.e., 640 nm laser irradiation, co-cultivation with *S*. 2973 or both of them) for 24 h. **(F)** Phenotypes of *S*. 2973 treated with different concentrations of ICG exposed to 2 W/cm^2^ of 808 nm laser for 5 min.

Aiming at alleviating hypoxia and enhancing the PDT effect, we tested the bio-oxygen pump *in vitro* first (Figure 3A). To ensure the viability of *S*. 2973 and sustainable O_2_ generation when used *in vivo*, a 640 nm red laser (Red) was chosen given the utilized spectrum of cyanobacteria and the penetrability of light toward tissue. Then red lasers with different intensity of 0, 0.1, 0.25, and 0.5 W/cm^2^ were tested. Density of 0.25 W/cm^2^ was found feasible as growth of *S*. 2973 was accelerated with the increasing laser density but was inhibited when it reached up to 0.5 W/cm^2^ (Figure 3B). In addition, correlated with the condition under normal light, longer irradiation time increased the growth of *S*. 2973 (Figure 3C). Meanwhile, O_2_ generation was demonstrated via detecting the dissolved O_2_ irradiated with the red laser of 0.25 W/cm^2^ (Figure 3D). Although no literature evidence of *Synechococcus*-derived cytotoxins or cytotoxicity was found (Klemenčič et al., 2017), we evaluated the cytotoxicity of *S*. 2973 to ensure its biosafety. As expected, cell viability assay and AM/PI staining assay didn’t find significant cytotoxicity from *S*. 2973 or red laser irradiation (Supplementary Figure S3 and Figure 3E), indicating the biosafety is in general assured. To further guarantee the biosafety when used *in vivo*, the eliminating effect caused by PDT was also tested. As illustrated in Figure 3F, complete inactivation of *S*. 2973 could be observed when treated with 20 μg/mL ICG after 5 min irradiation with 808 nm laser, indicating PDT could synchronously inhibit the tumor and eliminate *S*. 2973.

### Creation and Evaluation of the Controlled-Release Injectable Hydrogels Packaging With *S.* 2973 and MSN-ICG

**FIGURE 4:**
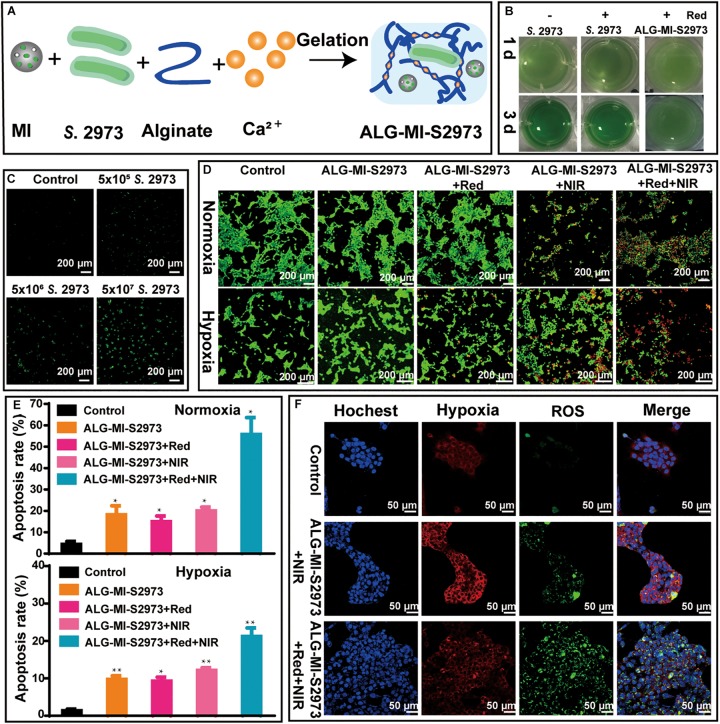
Construction and characterization of injectable hydrogels packing MSN-ICG and/or *S*. 2973. **(A)** The preparation of injectable hydrogels packing MSN-ICG and S. 2973 (i.e., ALG-MI-S2973). **(B)** Growth investigation of *S*. 2973 directly in BG11 or packaged in ALG-MI-S2973 cultivated under normal or 640 nm irradiation. **(C)** The ROS generation in 4T1 cells after treatment of ALG-MI-S2973 with different amounts of *S*. 2973. **(D,E)** AM/PI staining and cell apoptosis analysis of 4T1 cells treated with or without ALG-MI-S2973 under control, 808 nm irradiation or both 640 and 808 nm irradiation. **(F)** Measurements of hypoxia and ROS of normoxia 4T1 cells treated with or without ALG-MI-S2973 under control, 808 nm irradiation or both 640 and 808 nm irradiation. * *p* < 0.05, ** *p* < 0.01 compared with untreated group by Student’s *t*-test. MSN-ICG, indocyanine green-loaded mesoporous silica nanoparticles; ALG-MI-S2973, hydrogels packaging MSN-ICG and *S*. 2973.

To provide an oxygen pump for PDT, we created a combined system packing both *S.* 2973 and MSN-ICG into injectable hydrogels named ALG-MI-S2973 (Figure 4A and Table 1). As shown in Figure 4B, though slightly slower than the *S.* 2973 cultivated under normal condition with white light or red light (640 nm), *S.* 2973 can grow well in the hydrogel system. In addition, continuous release of ICG from MSN as long as 72 h could be detected due to the package of MSN and ALG (Supplementary Figure S2). Further, we treated 4T1 cells respectively with control and ALG-MI-S2973 to determine its effect *in vitro*. Mixture of 4T1 cells and ALG-MI-S2973 led to strong ROS signal orderly after 640 and 808 nm laser irradiation, suggesting the increasing O_2_ evolution generated from ALG-MI-S2973 with the increasing amount of *S.* 2973 (Figure 4C). Besides, as illustrated in Figures 4D,E, compared with PDT based on MSN-ICG, apoptosis rate of 4T1 cells based on ALG-MI-S2973 was decreased from ∼37 to 22% with only 808 nm irradiation under normoxia, indicating the slower release of ICG from ALG-MI-S2973 than that from MSN-ICG. Nevertheless, after cascaded irradiation with 640 nm for 5 min then following 808 nm excitation to induce the oxygen production and PDT sequentially, apoptosis rate of 4T1 cells based on ALG-MI-S2973 was dramatically increased up to ∼56%. More importantly, the new PODT system could significantly increase the effect under hypoxia (∼22% apoptosis rate) (Figures 4D,E), suggesting the feasibility of the photo-oxygen-dynamic oncotherapy *in vitro*. Consistently, hypoxia and ROS measurements for 4T1 cells under normoxia showed that the addition of red laser could significantly alleviate the hypoxia and promote the PDT due to the O_2_ evolution, compared with ALG-MI-S2973 without 640 nm irradiation (Figure 4F).

### Long-Term Biosafety Investigation of *S.* 2973 on Healthy Mice

**FIGURE 5:**
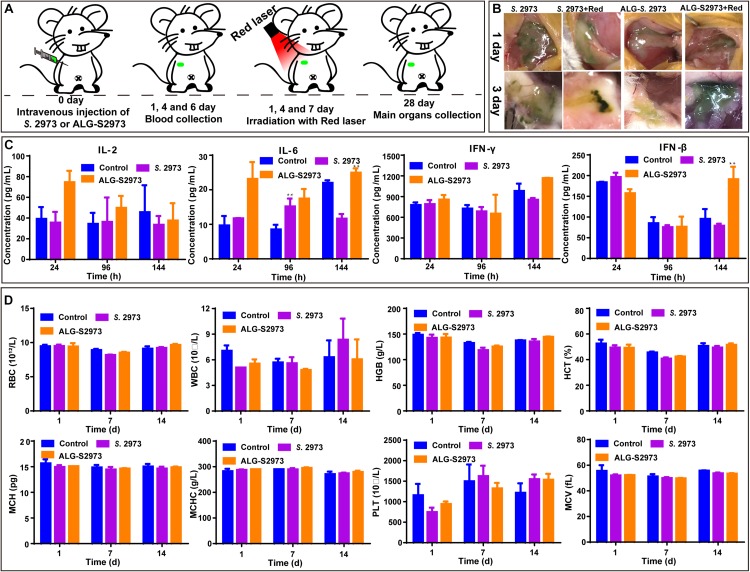
The *in vivo* long-toxicity evaluation of *S*. 2973 and ALG-S2973. Each group contained three mice. **(A)** The schedule of animal experiments for toxicity evaluation. **(B)** Photos of the injection site of BALB/c mice. **(C)** Detection of immune factors of mice with different treatments at the 1st, 4th, and 7th day. **(D)** Hematological index of the mice with different treatments at the 1st, 7th, and 21st day. * *p* < 0.05, ** *p* < 0.01 compared with untreated group by Student’s *t*-test. ALG-S2973, hydrogels packaging *S*. 2973.

The biosafety of ICG has been demonstrated previously by several independent studies (Bradley and Barr, 1968; Sauda et al., 1986). Therefore, before testing the PODT *in vivo*, long-term potential toxicity from *S*. 2973 on healthy mice was first investigated (Figure 5A). Detailly, female BALB/c mice were randomly divided into four groups (each group containing three mice) and two of them were subcutaneously injected with *S.* 2973 or ALG-S2973 (Table 1). All groups except control were irradiated with 640 nm laser on the 1st, 4th, and 7th day, the investigation of injection site suggesting the well survival of *S.* 2973 (Figure 5B). After 1, 4-, and 7-days’ injection, blood was collected to analyze the immune factors including IL-2, IL-6, IFN-γ and IFN-β (Figure 5C). Besides, blood was collected on the 1st, 7th, and 14th day after injection to conduct blood routine examination (Figure 5D). Finally, mice were sacrificed and main organs including heart, liver, spleen, lung and kidney were collected for pathological analysis (Supplementary Figure S4). Although certain properties like immune factor IL-6 showed slight change in mice group injected with *S.* 2973 or ALG-S2973 compared to the control group, we believe that it may be due to the limiting number of mice used in the biosafety evaluation experiments, which may lead to wide error bars. Although further biosafety experiments are certainly necessary in the future, our study found that consistent with the previous studies (Cohen et al., 2017), neither injection of *S.* 2973 nor ALG-S2973 caused any effect on immune factors, hematological index or main organs of mice, suggesting of in general good biosafety of both the *S.* 2973 and ALG-S2973 *in vivo*.

### Evaluation of the PODT *in vivo* Using 4T1 Tumor Model

**FIGURE 6:**
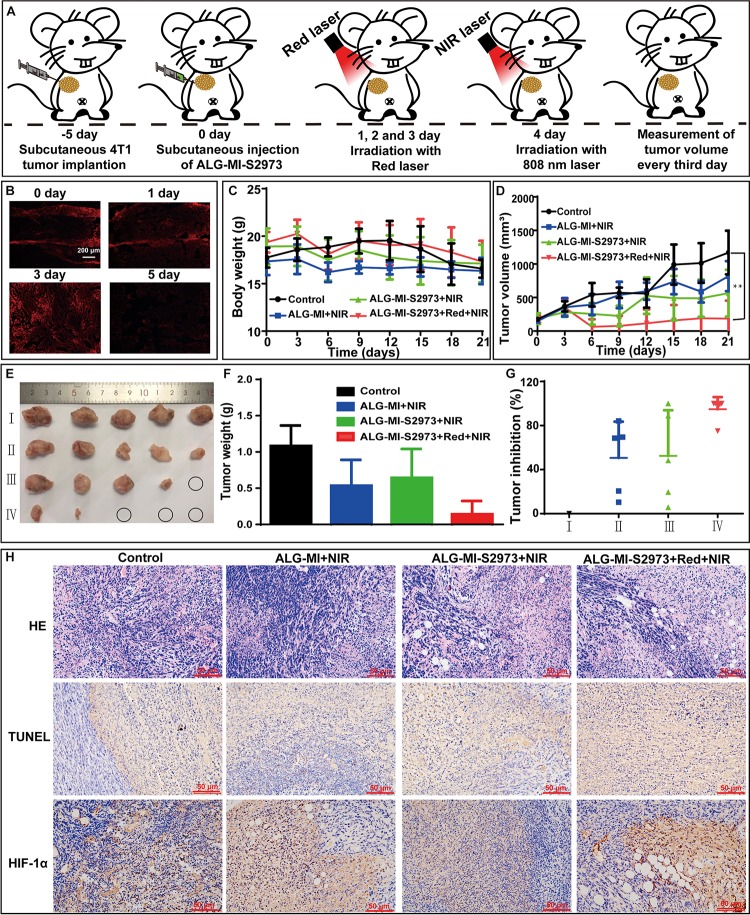
*In vivo* investigation of anti-tumor effects based on ALG-MI-S2973. Each group contained five mice. **(A)** Schematic diagram of animal experiments. **(B)** Viability measurement of *S*. 2973 in tumor sections using fluorescence imaging excited by 561 nm. **(C,D)** Body weight changes and tumor growth curves of control and 4T1 tumor-bearing mice with different treatments. ** *p* < 0.01 compared with untreated group by Student’s *t*-test. **(E,F)** Volumes and weight of tumors from 4T1 tumor-bearing mice with different treatments; **(G)** Tumor inhibition rates of different treatments. **(H)** H&E, TUNEL and immunohistochemical staining of HIF-1α protein in tumor tissues from 4T1 tumor-bearing mice with different treatments. ALG-MI, hydrogels packaging MSN-ICG; ALG-MI-S2973, hydrogels packaging MSN-ICG and *S*. 2973.

Finally, we investigated *in vivo* anti-tumor effects of ALG-MI-S2973 using the 4T1 tumor model (Figure 6A and Table 1). Detailly, female BALB/c mice were randomly divided into four groups and each group contained five mice. Besides the control group (Group I), one group were subcutaneously injected only with MSN-ICG (Group II) while the other two groups were subcutaneously injected with ALG-MI-S2973 (Group III and IV). After injection, 640 nm laser irradiation was conducted during the next 3 days to induce the oxygen generation then the 808 nm laser was used to induce the PDT on the 4th day for Group I and IV. While only 808 nm laser was used for Group II and III. Consistently, fluorescence imaging of *S.* 2973 in tumor tissue showed that they were accumulated in the first 3 days due to the radiation of red laser and were killed in the 5th day due to the PDT (Figure 6B). Body weight of 4T1 tumor-bearing mice with different treatments was recorded, suggesting no significant fluctuation during the process (Figure 6C and Supplementary Figure S5). In addition, the volume and weight of tumor tissue was recorded and pictured then the inhibition rate was calculated (Figures 6D–G). Though all treatments showed therapy effect of varying degrees, traditional PDT for group II could only achieve an average tumor inhibition rate at ∼50%. Excitingly, the addition of oxygen pump remarkably enhanced the PDT, showing amazing effect as the tumor tissue disappeared just from the 4th day for Group IV. The inhibition rate on 4T1 tumor were almost 100% for 4 of 5 mice in Group IV using photo-oxygen-dynamic therapy, reaching an average inhibition rate at ∼86%. Tumor tissue for 1 of 5 mice in Group III was also disappeared at last, which may be due to the original O_2_ production from *S*. 2973 just after injection. Finally, H&E and TUNEL demonstrated the apoptosis and elimination of tumor tissue in Group IV (Figure 6H). Furthermore, immunohistochemical staining of HIF-1α protein in tumor tissues showed that hypoxia induced factor HIF-1α was significantly decreased in Group IV due to the oxygen pump (Figure 6H).

## Discussion

All types of solid tumors, especially malignant solid tumors are subject to hypoxia, whose O_2_ levels are remarkably lower than their original tissue. Hypoxia affects tumor microenvironment and increases blood vessel formation, aggressiveness, metastasis, and resistance to treatment, which further restricts the oncotherapy effect like PDT. In this study, combining the bio-oxygenic cyanobacterium *S.* 2973 with traditional PDT based on ICG allowed us to avoid the endogenous and the PDT-induced hypoxia, achieving an inhibition rate up to almost 100% on 4T1 tumor cells. More importantly, the *in vivo* toxicity evaluation demonstrated the biosafety of the strategy, thus probably providing a promising oncotherapy strategy. Most recently, Zhou et al. (2019) reported a light triggered oxygen-affording engine for hypoxia-resistant oncotherapy via combining PDT with *Chlorella pyrenoidosa* (Zhou et al., 2019). Compared with the reported work, this study innovatively divided the growth of *S.* 2973 and the trigger of PDT via two lasers with different wavelengths, avoiding the killing of *S.* 2973 by PDT-induced ROS in the early stage thus ensuring the maximum oxygen generation. Meantime, the induction of PDT via 808 nm laser simultaneously cleaned the *S.* 2973, further guaranteeing the biosafety of the strategy. In addition, the construction of control released injectable hydrogels prolonged the functional time of the therapy, decreased the dose used and may improve the therapy effects further. Finally, compared with other oxygenic microorganism like *Chlorella*, mature genetic toolboxes have been developed for model cyanobacteria like *S.* 2973 (Sun et al., 2018), allowing for the further optimization of the cyanobacteria-based bio-oxygenic systems. Most Recently, Huo et al. (2019) first demonstrated the role of cyanobacterium *S. elongatus* PCC 7942 in promoting PDT effect. As a complementary study, our research also demonstrated the similar results using a more promising cyanobacterium *S*. 2973. Although the study here only evaluated the PODT using subcutaneous tumor model, the positive results suggested further efforts by increasing the oxygen generation of *S.* 2973, deleting its endotoxin for tail vein injection or even enabling its ability for synthesizing anti-tumor chemicals via synthetic biology may enhance the efficiency of the photo-oxygen-dynamic oncotherapy in the future.

## Conclusion

In this study, an injectable hydrogel packaging both an oxygen pump based on *S.* 2973 and ICG-loaded mesoporous silica nanoparticles of controlled-release was created and evaluated *in vivo*. The packaged hydrogel showed strong therapeutic effect on tumor tissues with only negligible side effect on healthy mice. The PODT presented here provided a promising therapy strategy against hypoxia-resistant tumor and may worth further modifications for therapeutic application.

## Data Availability Statement

All datasets generated for this study are included in the article/Supplementary Material.

## Ethics Statement

The animal study was reviewed and approved by the Experimental Animal Ethics Committee of Institute of Radiation Medicine, Chinese Academy of Medical Sciences.

## Author Contributions

TS, YZ, and CZ performed the experiments and wrote the manuscript. TS, YZ, CZ, HW, HP, JL, and ZL analyzed the data. LC, JC, and WZ designed the study and revised the manuscript.

## Conflict of Interest

The authors declare that the research was conducted in the absence of any commercial or financial relationships that could be construed as a potential conflict of interest.
