# Supplementary material for: Cyanobacteria-Based Bio-Oxygen Pump Promoting Hypoxia-Resistant Photodynamic Therapy
Source: Front Bioeng Biotechnol. 2020 Mar 24;8:237. doi: 10.3389/fbioe.2020.00237 (PMC7105637; doi:10.3389/fbioe.2020.00237)
Supplement: Supplementary file 1 [file Data_Sheet_1.docx]

Supporting information

**Cyanobacteria based bio-oxygen pump promoting hypoxia-resistant photodynamic therapy**

Tao Sun ^1,4,^ ^‡^, Yingying Zhang ^2,^ ^‡^, Chaonan Zhang ^2^, Hanjie Wang ^2^, Huizhuo Pan ^2^, Jing Liu ^2^, Zhixiang Li ^3,4,5^, Lei Chen ^3,4,5^, Jin Chang ^2, *^, Weiwen Zhang ^1,3,4,5, *^

*^1^* *Center for Biosafety Research and Strategy, Tianjin University, Tianjin, China; ^2^ School of Life Sciences, Tianjin University, Tianjin, China;* *^3^Laboratory of Synthetic Microbiology, School of Chemical Engineering & Technology, Tianjin University, Tianjin, China； ^4^ Frontier Science Center for Synthetic Biology and Key Laboratory of Systems Bioengineering, Ministry of Education of China, Tianjin, China；**^5^Collaborative Innovation Center of Chemical Science and Engineering, Tianjin, China*.

^†^ These two authors contributed equally to this paper.

* To whom all correspondence should be addressed:

Prof. Dr. Jin Chang

School of Life Sciences

Tianjin University, Tianjin, China

Tel: +86-22-27403906

E-mail: [jinchang@tju.edu.cn](mailto:jinchang@tju.edu.cn)

Prof. Dr. Weiwen Zhang

Laboratory of Synthetic Microbiology

School of Chemical Engineering & Technology

Tianjin University, Tianjin, China

Tel: +86-22-2740-6394; Fax: 0086-22-2740-6364;

Email: [wwzhang8@tju.edu.cn](mailto:wwzhang8@tju.edu.cn)


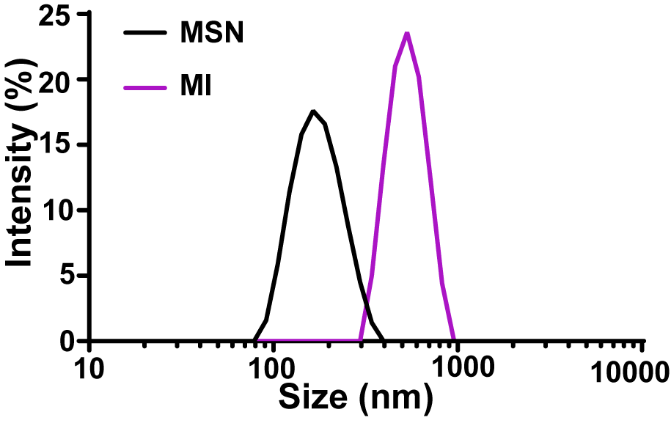


**FIGURE S1** Particle size distribution of MSN before and after ICG loading.

**FIGURE S2** Cumulative release of ICG from MSN-ICG and ALG-MI-*S*. 2973.

**FIGURE S3** Cell viabilities of 4T1 cells treated with control or *S*. 2973 with or without 640 nm red laser irradiation.


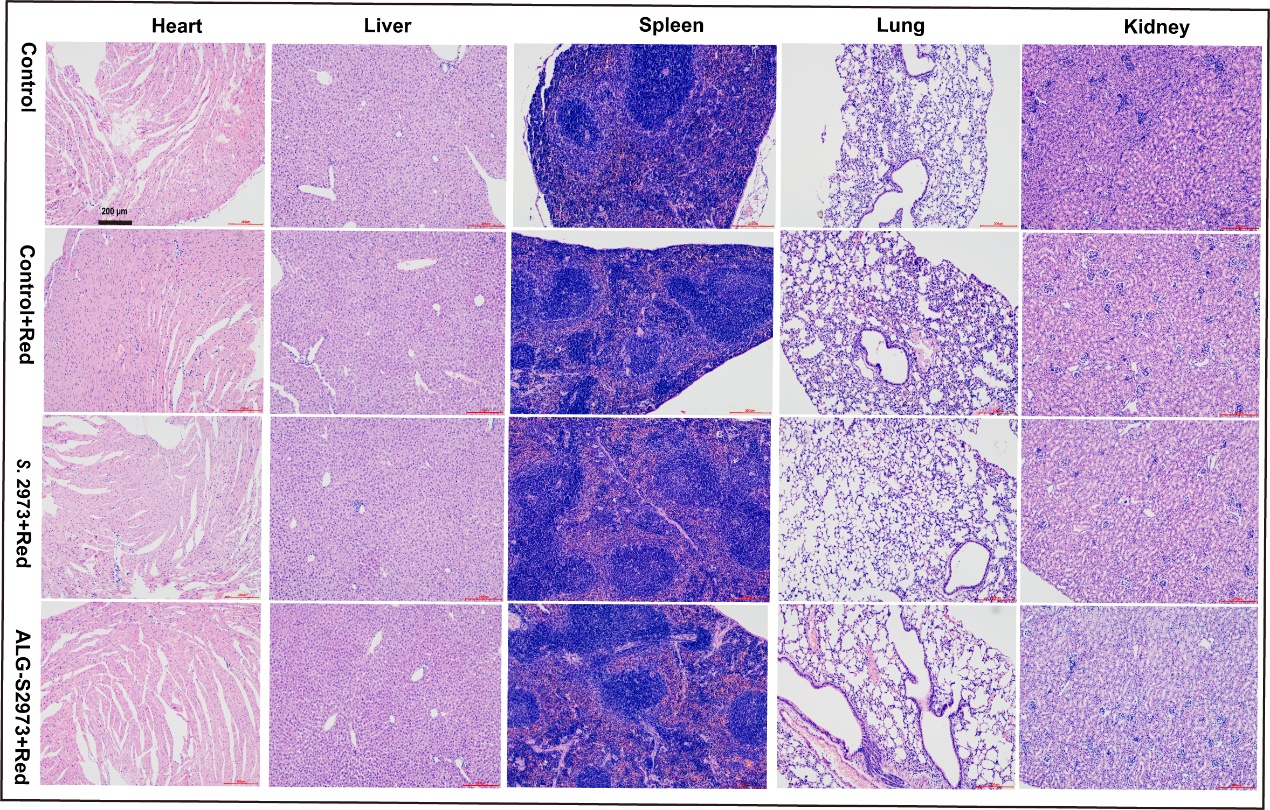


**FIGURE S4** H&E staining slices of main organs collected from mice after injection of *S*. 2973 after 28 days.


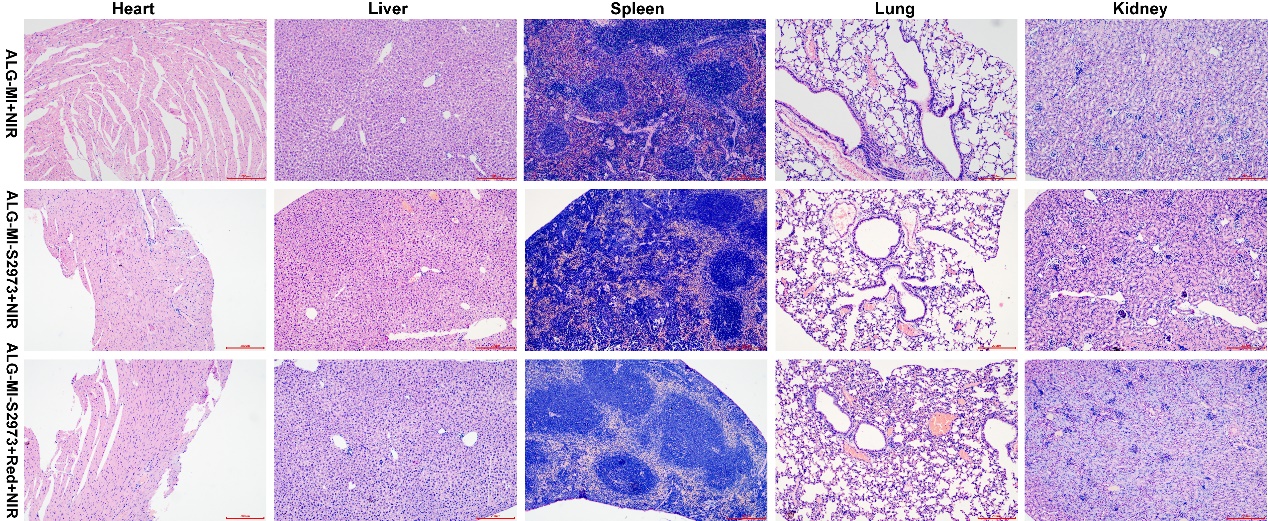


**FIGURE S5** H&E staining slices of main organs collected from mice after various treatments.
